# *Leishmania infantum* β-Tubulin Identified by Reverse Engineering Technology through Phage Display Applied as Theranostic Marker for Human Visceral Leishmaniasis

**DOI:** 10.3390/ijms20081812

**Published:** 2019-04-12

**Authors:** Lourena E. Costa, Patrícia T. Alves, Ana Paula Carneiro, Ana C. S. Dias, Patrícia T. Fujimura, Galber R. Araujo, Grasiele S. V. Tavares, Fernanda F. Ramos, Mariana C. Duarte, Daniel Menezes-Souza, Peter Briza, Fátima F. Briza, Eduardo A. F. Coelho, Luiz Ricardo Goulart

**Affiliations:** 1 Programa de Pós-Graduação em Ciências da Saúde: Infectologia e Medicina Tropical, Faculdade de Medicina, Universidade Federal de Minas Gerais, Belo Horizonte 30130-100, Minas Gerais, Brazil; lourena.costa@yahoo.com.br (L.E.C.); grasysv@hotmail.com (G.S.V.T.); fe.fonsecaramos@gmail.com (F.F.R.); marianacostaduarte1@gmail.com (M.C.D.); daniel.ufop@gmail.com (D.M.-S.); 2 Instituto de Genética e Bioquímica, Universidade Federal de Uberlândia, Uberlândia 38405-320, Minas Gerais, Brazil; patriciaterraalves@yahoo.com.br (P.T.A.); anapaulacarneirobio@yahoo.com.br (A.P.C.); diascarolina2004@hotmail.com (A.C.S.D.); dudafercoelho@pq.cnpq.br (P.T.F.); lrgoulart@ufu.br (L.R.G.); 3 Departamento de Patologia Clínica, COLTEC, Universidade Federal de Minas Gerais, Belo Horizonte 31270-901, Minas Gerais, Brazil; galber.araujo@gmail.com; 4 Department of Molecular Biology, University of Salzburg, 5020 Salzburg, Austria; dudafercoelho@yahoo.com.br (P.B.); dudaferc@cnpq.br (F.F.B.); 5 Department of Medical Microbiology and Immunology, University of California-Davis, Davis, CA 95616, USA

**Keywords:** phage display, β-tubulin protein, *Leishmania infantum*, scFv antibody, diagnosis, vaccine

## Abstract

Two *Leishmania infantum* mimotopes (B10 and C01) identified by phage display showed to be antigenic and immunogenic for visceral (VL) and tegumentary (TL) leishmaniasis; however, their biological targets in the parasites have not been identified. The aim of the present study was to investigate the native antigens expressing both mimotopes, and to use them in distinct immunological assays. For this, a subtractive phage display technology was used, where a combinatorial library of single-chain variable fragments (scFv) was employed and the most reactive monoclonal antibodies for each target were captured, being the target antigens identified by mass spectrometry. Results in immunoblotting and immunoprecipitation assays showed that both monoclonal scFvs antibodies identified the β-tubulin protein as the target antigen in *L. infantum*. To validate these findings, the recombinant protein was cloned, purified and tested for the serodiagnosis of human leishmaniasis, and its immunogenicity was evaluated in PBMC derived from healthy subjects and treated or untreated VL patients. Results showed high diagnostic efficacy, as well as the development of a specific Th1 immune response in the cell cultures, since higher IFN-γ and lower IL-10 production was found.

## 1. Introduction

Leishmaniasis is an infectious disease caused by protozoan parasites of the genus Leishmania, through the bite of infected phlebotomine sand flies [1]. Among clinical manifestations, visceral leishmaniasis (VL) is caused by *Leishmania infantum* (=*L. chagasi*) and *L. donovani* species, while tegumentary leishmaniasis (TL) is caused mainly by *L. major* and *L. tropica* species in the Middle East and central Asia, and by *L. braziliensis, L. amazonensis* and *L. mexicana* in the Americas [2].

Brazil is responsible by about 90% of VL cases, with the *L. infantum* species being the main parasite species responsible for the disease in dogs and humans [3]. Prophylactic measures have not been efficient to control this disease, and the available antileishmanial drugs present problems regarding their toxicity and/or high cost [4]. Therefore, the development of an effective vaccine could be considered as a strategy to control the spread of the disease. However, there is no an available vaccine against human VL, although some candidates have been well-evaluated in experimental trials using mice and dogs [5].

We have previously identified two mimotopes through the phage display technology, which mimic epitopes of *Leishmania infantum* antigens, being expressed in the B10 and C01 phage clones [6]. These molecules were successfully tested as vaccine candidates against murine VL, where the induction of, and maintained after challenge. Both phages were successfully protective against the *L. infantum* infection in BALB/c mice, and a specific Th1 immunity primed by high levels of IFN-γ, IL-12 and GM-CSF was observed before and after challenge [6]. In addition, a heterologous protection was also reached when mice were infected with *L. amazonensis* [7]. The B10 and C01 clones were also shown to present a high accuracy for the serodiagnosis of canine and human VL [8], since the phages and synthetic peptides showed high sensitivity and specificity values for the serodiagnosis of the disease. However, in despite of the high number of experiments using these molecules, the target antigens in *Leishmania* expressing B10 and C01 mimotopes (LSFPFPG and FTSFSPY, respectively) were still not identified.

The diagnosis of VL has been based on clinical evaluation and the application of distinct laboratory strategies, such as parasitological and immunological methods [9,10]. Parasitological exams, such as microscopy to identify amastigote forms in organ aspirates, although considered highly specific, present problems regarding their sensitivity. In addition, they require expertise by technicians and sample collection is an invasive procedure, thus limiting the diagnostic efficacy. Immunological methods have been also employed; however, problems related with the sensitivity and/or specificity of the antigens used in the tests are registered [11,12]. Thus, it is necessary to search for new candidates that will serve to design diagnostic systems with higher degree of sensitivity and specificity than current methods, such as the refinement of use of new antigens, such as recombinant proteins [13,14,15], synthetic peptides [16,17], polypeptide-based chimera [18,19] and phage clones [8,20].

The present study was developed to identify antigen targets in *L. infantum* parasites capable of expressing the B10 and C01 epitopes, by means of a reverse engineering process selecting monoclonal antibodies specific to those two mimotopes, through subtractive phage display technology. For this, a combinatorial library of single-chain variable fragments (scFv) was used, and the most reactive antibodies for each target were captured, being the native antigens identified by mass spectrometry. Results showed the recognition of the unique target antigen in parasites, namely β-tubulin, as expressing both epitopes. Then, the recombinant protein was cloned, purified and evaluated in immunological assays in the human VL.

## 2. Results

### 2.1. Characterization of scFv Antibody Clones

**Figure 1:**
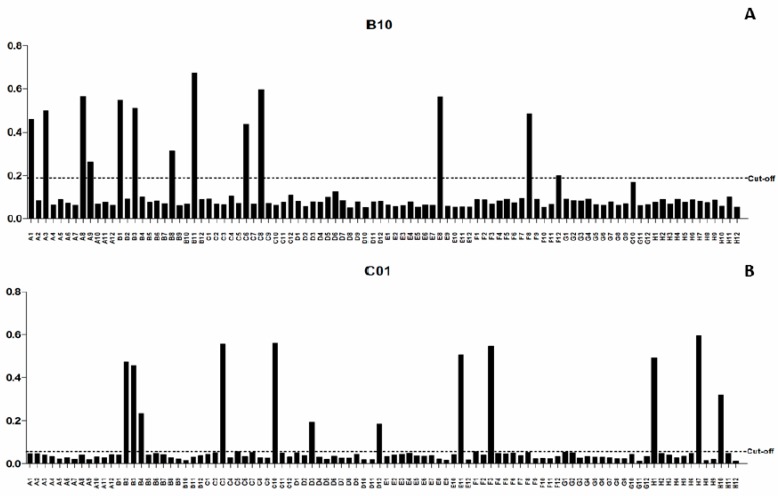
Evaluation of the soluble scFv expression after IPTG induction in the selected clones. The presence of soluble scFv fragments was evaluated after induction by IPTG using an anti-HA antibody and ELISA method. The scFv clones secreted anti-B10 phages were in a total of 13 (**A**) and anti-C01 in a total of 12 (**B**). In the *x*-axis we have the 96 possible clones secreted, in the *y*-axis we have the absorbance at 492 nm of the induced solution; the cut-offs were calculated based on the mean of the negative controls (clones that were not expressed) more or less two times the (SD) standard deviation (**A**,**B**).

**Figure 2:**
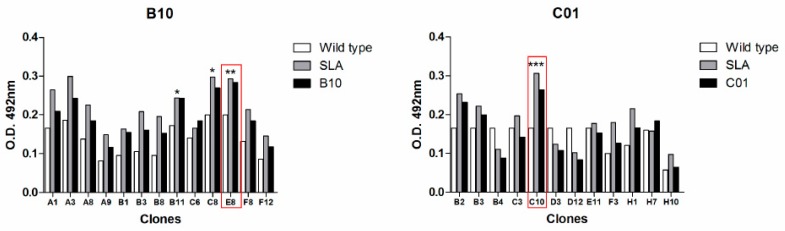
ELISA screening to identify the specific scFv antibody fragments. Three ELISA’ from each clone were performed. The scFv clones were reacted against B10 and C01 phages, *L. infantum* SLA and wild-type phage. The scFv-E8 and scFv-C10 clones showed higher reactivity to B10 and C01 phages, respectively. The scFv-E8 and scFv-C10 clones were selected due to the best absorbance ratio between the target and the controls, demonstrating better antibody specificity and a most significant *p*-value (* *p* < 0.05; ** *p* < 0.01, and *** *p* < 0.005). The *x*-axis have the clones effectively secreted anti-B10 or C01 and on the *y*-axis shown the absorbance (492) nm of the samples in ELISA.

**Figure 3:**
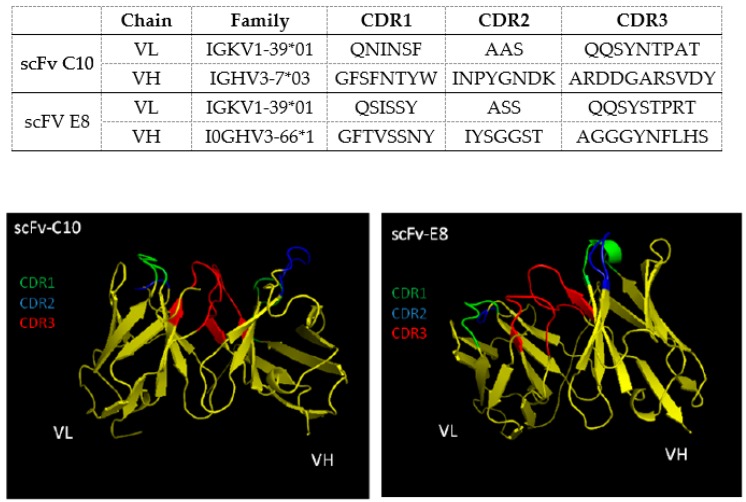
Characterization of the selected scFv clones. The amino acid sequences and three-dimensional structures of scFv-E8 and C10 clones, the CDR residues, variable light-chain (VL) and the variable heavy-chain (VH) domains are shown.

After the bio-selection cycles, clones were grown in 96 deep-well plates, and expression induction of scFv antibodies was evaluated by ELISA. Among the pre-selected scFv clones, 13 and 12 were isolated as being specific to B10 and C01 clones, respectively (Figure 1). The specificity of the scFv was tested against B10, C01, wild-type M13 and *L. infantum* SLA. The scFv-E8 anti-B10 phage demonstrated higher reactivity to B10 phage and *L. infantum* SLA (Figure 2). Similar results were obtained using the scFv-C10 anti-C01 phage against C01 clone. The analysis of the nucleotide sequences led to the identification from heavy- and light-chain variable regions of the scFv clones, and their predicted three-dimensional structures are shown (Figure 3).

### 2.2. Identification of Target Protein in Leishmania Parasites

**Figure 4:**
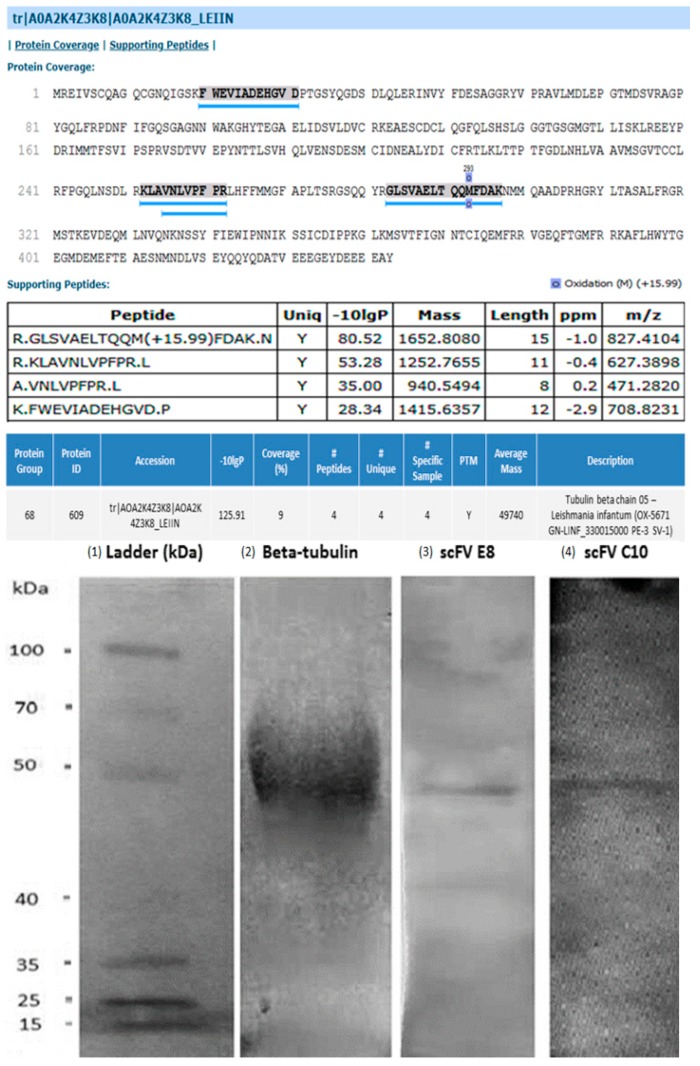
Identification of *Leishmania* β-tubulin protein. The target protein was identified by mass spectrometry (**top**) and immunoblotting reaction (**bottom**) against the selected monoclonal antibodies. Immunoblotting experiments using the scFv-E8 and scFV-C10 antibodies were performed for confirmation of the target protein. SDS-PAGE gel followed by Western blot assay confirmed that both monoclonal antibodies recognized the β-tubulin protein, presenting a molecular weight of 49.8 kDa. (1) Protein molecular weight marker (in kDa). (2) The recombinant β-tubulin protein reacting against the anti-Histidine monoclonal antibody. The reaction of the scFv-E8 and scFV-C10 antibodies with the purified protein is shown (in (3) and (4), respectively).

**Figure 5:**
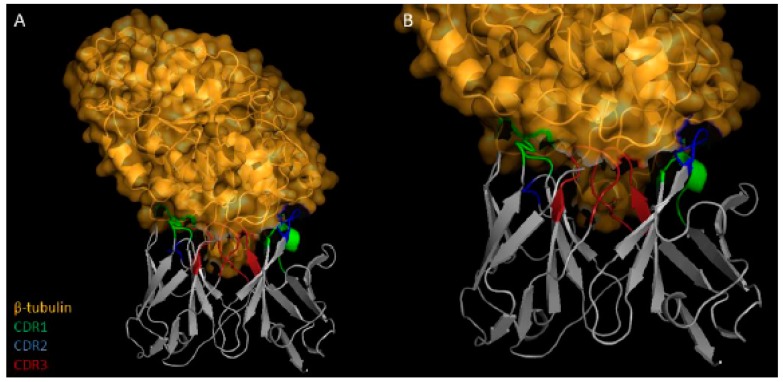
Three-dimensional analysis of the interaction between antibodies and targeted protein. Results (**A**,**B**) showing the regions of interaction between the scFv E8 (gray) and β-tubulin (orange) are shown, as well as structures from CDR regions of the scFv antibodies associated to β-tubulin protein are shown.

An immunoprecipitation assay, mass spectrometry and immunoblotting were carried out to identify the *L. infantum* protein that was specific for the scFv antibodies. Parasite proteins were immune-precipitated and β-tubulin was selected as the target protein. Afterwards, immunoblotting assays confirmed also the binding of the both scFv antibodies to β-tubulin, with both clones recognized it as the common antigen, presenting an estimated weight of 49.8 kDa (Figure 4). A 10% SDS-PAGE gel showing the purified recombinant protein was performed, and it is shown (Figure S1). Docking assays were performed between the scFv and β-tubulin, in order to determine possible binding sites by using the in silico prediction tools and the predicted 3D structure of interaction between scFv and *Leishmania* β-tubulin (Figure 5). The formed complex was analyzed by the PyMOL tool and residues were identified as making polar contacts between scFv and protein. The Ser^30^, Ser^56^, Gln^27^ present in the scFv-E8 clone interact with Glu^22^, Glu^281^, Thr^33^ of β-tubulin, respectively, while Ser^30^ and Gln^27^ are residues from CDR3 light chain. However, due to prediction problems in the scFv structure, no interaction between scFv-C10 clone and β-tubulin was detected.

### 2.3. Diagnostic Potential and Immunogenicity of Recombinant β-Tubulin Protein

**Figure 6:**
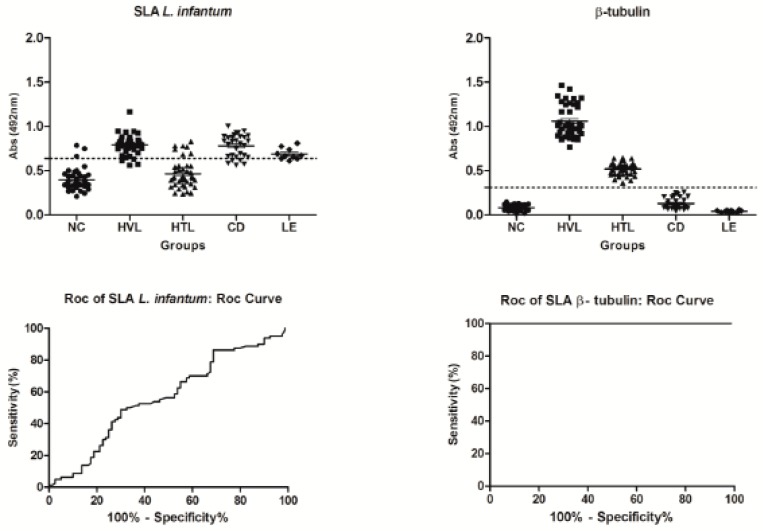
Serological reactivity of recombinant β-tubulin protein and *L. infantum* SLA in serum samples from patients and controls. ELISA assays using human serum samples from uninfected resident individuals in an endemic area of leishmaniasis (NC, *n* = 40), visceral leishmaniasis patients (HVL, *n* = 40), tegumentary leishmaniasis (HTL, *n* = 40), Chagas’ disease (CD, *n* = 30) and leprosy (LE, *n* = 10) were tested using total protein extract of *L. infantum* and the recombinant β-tubulin. In the *x*-axis we have the experimental groups of the patients and in the *y*-axis the absorbance (492) nm of the samples in the ELISA.

**Figure 7:**
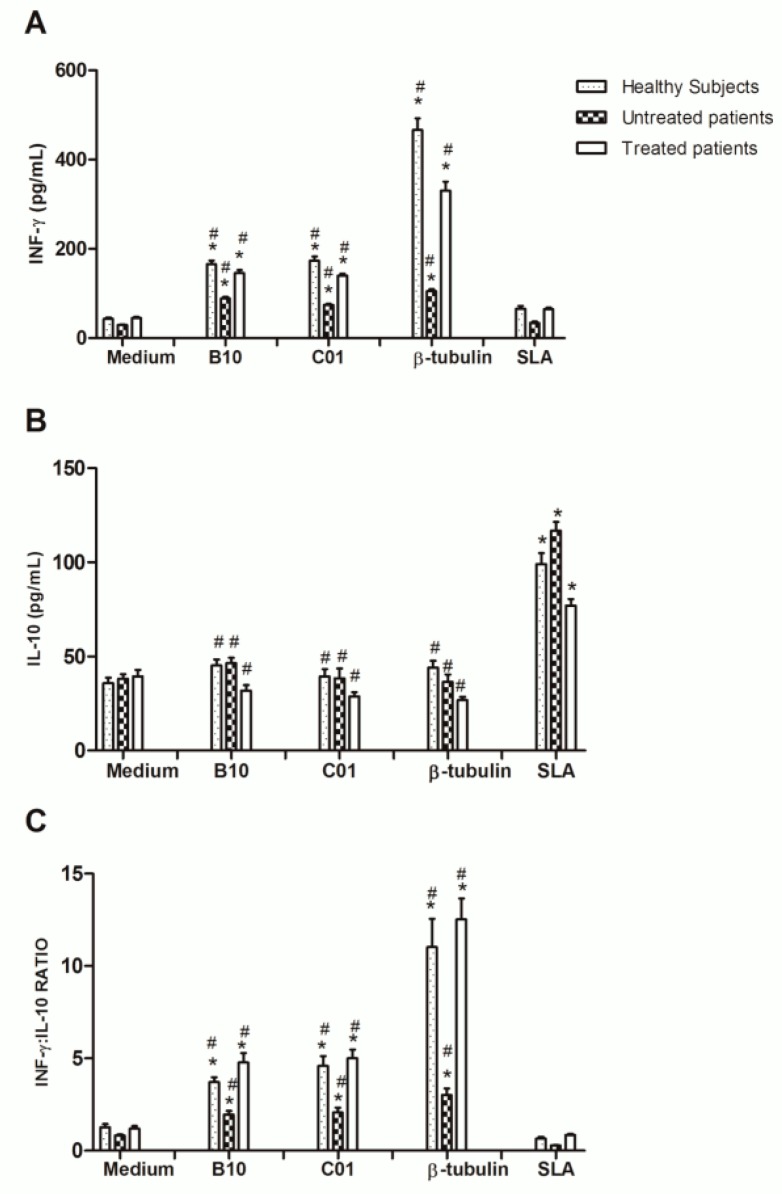
IFN-γ and IL-10 production in stimulated PBMC cultures derived from treated and untreated VL patients and control subjects. Peripheral blood mononuclear cells (PBMCs) were purified from blood samples from VL patients (*n* = 10), which were collected before and six months after treatment, as well as from healthy subjects living in an endemic area of disease (*n* = 10). Cells (10^7^) were non-stimulated (medium) or stimulated with B10, C01 or irrelevant (NRP) phage clones (10^10^ molecules, each), with the B10 or C01 peptides (10 µg/mL each) or with β-tubulin or *L. infantum* SLA (10 and 25 µg/mL, respectively), for 5 days at 37 °C in 5% CO_2_. Cell supernatant was collected and the specific IFN-γ and IL-10 production was measured by capture ELISA (in **A** and **B**, respectively). The ratio between the IFN-γ and IL-10 levels was calculated and is also shown (in **C**). Bars indicate the mean ± standard deviation of the groups. * indicates statistically significant difference in relation to the medium (*p* < 0.0001). # indicates statistically significant difference in relation to the SLA *L. infantum* stimulus (*p* < 0.0001).

The antigenic potential of the β-tubulin protein for the serodiagnosis of human VL was evaluated by using about 160 human sera collected in Brazil (Figure 6). Results showed that β-tubulin presented good performance to detect positive samples, since sensitivity and specificity values were 100% in both cases. ROC curves showed accuracy of 100%, with an AUC of 0.9979 and confidence interval of 95%, ranging from 0.9541 to 1.000 (*p* < 0.0001). In addition, the immunogenicity of B10 and C01 clones was evaluated in human cells, when cell supernatants were collected and the cytokine production was evaluated by capture ELISA. Results showed that clones, as well as their synthetic peptides induced higher IFN-γ and lower IL-10 levels in PBMCs collected from untreated and treated VL patients and healthy subjects. The β-tubulin protein was also used, and results showed higher IFN-γ/IL-10 ratio in all groups, thus demonstrating the potential immunogenic of this protein in human cells (Figure 7).

## 3. Discussion

We have previously shown that two phage clones, B10 and C01, which were recognized by antibodies in VL dogs sera, were protective against *Leishmania* infection and diagnostic markers for VL [6,7,8]. In fact, besides of their role as epitope-based vaccines to induce Th1 immunity and protection against *L. infantum* and *L. amazonensis* infection; these molecules showed also a satisfactory performance for the serodiagnosis of human and canine disease [6,7]. In the present study, we have performed a reverse engineering process by phage display using a combinatorial antibody library, aiming to identify target antigens in *L. infantum* capable of expressing the B10 and C01 mimotopes. The selection of combinatorial scFv antibodies against each phage led to the identification of two highly reactive antibodies (E8 and C10), which were capable of capturing the β-tubulin protein in immunoprecipitation reactions, as evidenced by mass spectrometry, immunoblotting and ELISA assays. This fact can be explained due to the recognition of different epitopes and their ligation sites in the same protein structure. Interestingly, predicted residues defined a conformational epitope of β-tubulin, which is in direct contact with residues of the hypervariable loops of the CDR3 light chain (CDR-L3), corroborating with the notion that kappa and lambda light chains of the CDR-L3 may present different roles in the adaptive immune response [21].

Tubulins are members of a family of globular proteins responsible for the cell cytoskeleton [22]. In Leishmania, they interact forming microtubules, which play an important role not only in cell structure and division, but also in keeping the shape and motility of the parasites [23]. They represent housekeeping proteins found in the cytoplasm and nucleus of parasites, being abundantly expressed in both promastigote and amastigote forms, and considered as important components of leishmanial flagella [24]. β-tubulins have been previously described as Th1 cell-stimulating agents; then demonstrating their potential to be evaluated as vaccine candidates against leishmaniasis [25,26].

The infection in humans with Leishmania parasites can results in distinct clinical manifestations, depending on their ability to evade the immune response of the host by means of a well-structured orchestra of host-parasite interactions [27]. Modulations of the humoral and cellular responses in asymptomatic patients and in healthy subjects living in endemic region of disease, where the most of the subjects develop a Th1 response based on the production of cytokines, such as IFN-γ and IL-12; support the feasibility to develop a human vaccine, such as by testing new candidates in in vitro and/or in vivo experiments using human cells [28,29]. In this study, B10 and C01 phages, as well as the recombinant β-tubulin protein, were used to stimulate PBMC from treated and untreated VL patients and healthy subjects, aiming to evaluate the immunogenic profile induced by these molecules. From the data obtained, we postulate that treated VL patients and healthy individuals exhibited a similar immune profile, since higher IFN-γ and lower IL-10 levels were found in the cell supernatant after the stimulation suing the protein and phage clones.

The limitations found by the use of conventional parasitological and immunological techniques to diagnose VL have led to an increasing exploration of new targets to develop an accurate diagnosis, aiming to detect the disease in infected subjects; but not in these non-infected individuals but living in endemic areas of VL [30]. In addition, technological tools have been employed with the purpose to identify new molecules [21,31,32]. As an example, a recent immunoproteomic study performed in *L. braziliensis* promastigotes and amastigotes employing serum samples of TL patients allowed to the identification of antigenic proteins in the parasites. In this context, one could speculate about the diagnostic potential of these molecules. Between the identified targets, β-tubulin was showed to be recognized by antibodies in sera from both cutaneous and mucosal leishmaniasis patients [33]. In this study, the protein was cloned and its recombinant version was used in ELISA assays for the diagnosis of the disease, and results showed sensitivity and specificity values of 100% in both cases; thus demonstrating the biological potential of this protein to be used in the serodiagnosis of disease.

The diversity created by gene recombination and somatic hypermutation makes protein sequencing of monoclonal antibodies a challenge [34]. Mass spectrometry-based sequencing will provide a single unambiguous sequence for variable domains, which can result in the need for empirical testing of candidate sequences, sometimes iteratively, to identity one that can replicate the activity of the parental antibody [35]. Here, we describe an approach to antibody protein sequencing using the phage display technology and a combinatorial library of sequences followed by mass spectrometry, aiming to investigate the native targets probable to express B10 and C01 mimotopes in *Leishmania*. This strategy can be considered innovative, and could well help in other studies where phage display technology is employed, when mimotopes are selected as biological targets against distinct diseases, allowing the identification of the native antigens expressing these epitopes in microorganisms.

In conclusion, we have applied a reverse engineering technology through phage display selection using a combinatorial scFv antibody library against phage clones expressing two target mimotopes, which led us to the identification of *L. infantum* β-tubulin as a potent theranostic antigen on leishmaniasis. These immunogenic domains of β-tubulin may be used in vaccine formulation for protection against *Leishmania* spp. infection, as well as differential components for the serodiagnosis of disease in *L. infantum*-infected dogs and humans.

## 4. Materials and Methods

### 4.1. Ethics Statement

Experiments were performed in compliance with the national guidelines, as set forth by the Institutional Animal Care (Law number 11.794, 8 November 2009), and the Committee on the Ethical Handling of Research Animals from the Federal University of Minas Gerais (UFMG), who approved this study under protocol number 225/2017 (25 September 2017).

### 4.2. Parasites

Experiments were carried out using the *L. infantum* (MOM/BR/1970/BH46) strain. Parasites were grown at 24 °C in Schneider’s medium (Sigma-Aldrich, St. Louis, MO, USA), supplemented with 20% heat-inactivated fetal bovine serum (FBS; Sigma-Aldrich), 20 mM l-glutamine, 200 U/mL penicillin, and 100 µg/mL streptomycin, pH 7.4. The Leishmania antigenic extract (SLA) was prepared from stationary promastigotes, after few passages in liquid culture, as described elsewhere [36]. Briefly, 2 × 10^8^ promastigotes per mL, in a volume of 5 mL, were washed 3 times in 5 mL of cold sterile phosphate-buffered saline (PBS 1×). After five cycles of freezing and thawing, the suspension was centrifuged at 8000× *g* for 20 min at 4 °C, and the supernatant containing SLA was collected in 500 μL aliquots and stored at −70 °C, until use. The protein concentration was estimated by Bradford method [37].

### 4.3. Phage Display for Selection of B10 and C01 Mimotopes-Specific scFv Antibodies

For the selection of the specific monoclonal antibodies, a scFv phage library was developed according described [38]. Briefly, total RNA samples were obtained from leukocytes in peripheral blood samples collected from 25 healthy individuals, when RNA content was extracted by using TRIzol reagent and mixed for the cDNA synthesis. Afterwards, the heavy- and light-chain variable regions (VH and VL) were separately amplified by PCR using primers described elsewhere [39]. The amplified VH and VL segments were purified from the agarose gel after electrophoresis, pooled and linked through a series of overlap PCR steps to give the final scFv products in VL-linker-VH format, with GGSSRSS linker [40]. The scFv gene products were purified and ligated into the pComb 3× vector, digested with SfiI restriction enzyme, and followed by electroporation into XL1-Blue electrocompetent cells (Stratagene, La Jolla, CA, USA). Colonies were grown on agar plates with carbenicillin (100 mg/mL) and 40 mM glucose. Afterwards, colonies were counted to determine the library size, as described elsewhere [41]. The scFv antibody fragments displayed on the surface of the filamentous phage was performed by rescuing with VCS-M13 helper phage (Stratagene), as previously described [42]. The amplified fragments for the variable regions of the heavy- (VH) and light-chain (VL) gene repertoire corresponded to the expected sizes of 400 and 350 base pairs, respectively. The resulting VH and VL fragments were assembled into scFv inserts by overlapped PCR reactions, allowing the random association of heavy and light chains to maximise the combinatorial re-assortment. The resulting library size was 1 × 10^6^ clones. The variable regions were derived from nine different V gene families, including three VH gene families (VH1, 3 and 4) and six VL subgroups (Vk 1, 2 and 3 and Vl 1, 2, 4, 5 and 6); demonstrating that scFv fragments were distributed across the full repertoire of antibody germ-line genes. Then, the bio-selection cycles of scFv antibody clones specific to the mimotopes expressed in B10 and C01 phages were performed as described [38,43,44]. Initially, beads (Dynabeads^®^M-270 Epoxy, Invitrogen Life Sciences, Waltham, MA, USA) were linked to each phage clone (B10, C01, wild-type, and irrelevant phages; 10^10^ molecules each), according the manufacturer’s instructions. To screen the scFv library and select clones, beads coupled with the wild-type and irrelevant phages (20 µL) were placed sequentially into a microtube and incubated with the scFv library (50 µL) for 1 h each, respectively, when scFv clones that interacted with wild-type and irrelevant phages were removed. Next, the supernatant was collected by using a magnetic apparatus and transferred to two new tubes containing beads coupled with the target B10 and C01 mimotopes, and the incubation for 1 h at 37 °C was performed. The supernatant was discarded, and bound phages were washed 10 times with PBS/0.05% Tween 20; being then eluted with 100 μL of 100 mM glycine-HCl, pH 2.2, for 10 min at room temperature (RT), and the solution pH was neutralized by adding Tris base (2 M, pH 9.1). Then, selected clones were transferred to a XL1-Blue culture for amplification and titration. Clones were extracted from *E. coli* XL1-Blue bacterial cells infected in the bio-selection cycles for further transformation in *E. coli* TOP10 [44]. Aliquots of the transformed cells were plated on LB medium containing carbenicillin (100 μg/mL), and plates were incubated for 16 h at 37 °C. After, colonies were transferred to a well in a deep well plate containing 1 mL of SB medium containing carbenicillin (100 μg/mL) and 2% glucose solution (2 M). After centrifugation, the expression of soluble scFv clones was induced by incubation with isopropyl β–D thiogalactopyranoside (IPTG; Sigma, USA) to a final concentration of 2.5 mM and carbenicillin (100 μg/mL) for 16 h at 30 °C. The plates were centrifuged at 4000× *g* for 15 min at 4 °C, and the supernatant containing the soluble scFv clones was transferred to another 96-well plate and stored at 4 °C.

### 4.4. ELISA to Evaluate the scFv Expression

For the analysis of scFv clones’ expression, an ELISA experiment was performed. For this, supernatants containing soluble scFv antibodies were incubated in high affinity microplates, and the incubation was processed for16 h at 4 °C. Then, plates were washed with washing solution (PBS 1× and Tween 20 0.05%) and blocked with PBS 1× and BSA 3%, for 1 h at 37 °C, followed by two washing using only PBS 1× and three washes with washing solution. After, plates were incubated with anti-HA horseradish peroxidase-conjugated antibody (Roche, Switzerland), which was 1:2500 diluted in PBS 1× and BSA 0.3%, for 1 h at 37 °C. Then, plates were washed again as described earlier, and reactions were developed by adding 30% H_2_O_2_, 2 mg of ortho-phenylenediamine, and 0.1 M citrate-phosphate pH 5.0; when the absorbance was read in an ELISA reader (Titertek Plus, Flow Laboratories, Pforzheim, Germany) at 492 nm.

### 4.5. Analysis of Selected Clones’ Specificity

For the analysis of specificity of the scFv antibody clones, polystyrene microplates (Nunc MaxiSorp, St. Louis, MO, USA) were coated with 1 × 10^11^ plaque-forming unit (PFU) or *L. infantum* SLA (2.0 μg/well) for 16 h at 4 °C. A wild-type clone (1 × 10^11^ PFU per well) was used as control. Plates were washed 3 times with washing solution, and blocked with 100 μL from a solution composed by PBS 1× and BSA 5%, for 1 h at 37 °C. Then, 50 μL of scFv supernatants were added, and the incubation occurred for 2 h at 37 °C. The anti-HA peroxidase-conjugated antibody (1:2500 diluted) was added, and the incubation occurred for 1 h at 37 °C. After washing, reactions were developed according described above, and optical densities were read in an ELISA reader at 492 nm.

### 4.6. Purification of Selected scFv Clones and Identification of the Native Antigen

For the purification of the scFv antibody-display phage clones, His-tagged scFv antibody fragments were immobilized in a metal (Nickel) affinity chromatography column (HisTrapTM HP, GE Healthcare Life Sciences, Pittsburgh, PA, USA) using a HPLC system (AKTATM purifier), according to the manufacturer’s instructions. The purified scFv clones were concentrated using a 10 kDa Amicon apparatus (Millipore, St. Louis, MO, USA), and the protein concentration was estimated as described elsewhere [45]. Extraction with Triton X-114 was used to remove endotoxins from the purified scFv clones. For this, Triton X-114 was added to a concentration of 1%, and the sample was shaken for 30 min at 4 °C, and incubated for 10 min at 37 °C. Subsequently, the sample was centrifuged at 20,000× *g* for 10 min at 25 °C, in order to form two phases and the upper phase containing the scFv antibody was collected. The technical procedure was repeated three times, when the limulus amebocyte lysate commercial kit (Lonza, Verviers, Belgium) was used to evaluate the endotoxin level in the purified samples. The purified scFv clones showed near 96% purity and reached a yield of approximately 30.0 mg/L). To identify the corresponding native antigens in Leishmania for each obtained scFv, selected clones were coupled to beads, and 100 μL of mouse anti-His mAb Mag Beads (GenScript Transforming Biology Research, Piscataway, NJ, USA) were added with 100 μL of purified scFv (E8 and C10) clones, according to the manufacturer´s instructions. After washing, *L. infantum* SLA (1 mg/mL) was added and, after incubation and additional washings, 20 μL of the sample buffer were added, and the incubation for 5 min at 100 °C was performed. The immune-precipitated molecules with the monoclonal antibodies were eluted from beads by using a magnetic apparatus, and they were submitted to mass spectrometry (CID-MS/MS).

### 4.7. DNA Sequencing and Bioinformatics Assays

The sequencing was performed in heavy- and light-chain variable region genes of selected antibodies, by using the MegaBACE 1000 sequencer (Molecular Dynamics, Sunnyvale, CA, USA). Reactions were developed employing the DyEnamic ET Dye Terminator Cycle Sequencing Kit (GE Healthcare, USA), following the manufacturer’s instructions. The nucleotide sequence was determined using MMB4 (5′-GCTTCCGGCTCGTATGTTGTGT-3′) and MMB5 (5′CGTTTGCCATCTTTTCATAATC-3′) primers, and antibody variable domain sequences and V gene families were analyzed by the Ig-BLAST (http://www.ncbi.nlm.nih.gov) and VBASE2 (http://www.vbase2.org) programs [46]. The three-dimensional prediction of scFv was performed by Raptorx program (http://raptorx.uchicago.edu/), and scFv antibody interaction with the target protein (β-tubulin; accession number (UniProtKB): P21148) was evaluated by the Pathdock program (http://bioinfo3d.cs.tau.ac.il/PatchDock/). Structural representations were rendered by the PyMOL Molecular Graphics System program (http://www.pymol.org).

### 4.8. Cloning, Expression and Purification of Recombinant β-Tubulin Protein

The region codifying the β-tubulin (XP_001468164.1) gene was cloned from the *L. infantum* genomic DNA by using the (GCTAGCATGCGTGAGATCGTTTCCTG) and (5′GAGCTCCTAGTAGGCCTCCTCCTC) primers. The PCR amplified products were analyzed on agarose gels, excised from the gels, purified, digested with restriction enzymes and ligated to the pET28a-TEV vector. The recombinant plasmid was introduced to *Escherichia coli* BL21 Arctic Express (DE3) cells (Agilent Technologies, Santa Clara, CA, USA) by electroporation using a MicroPulser Electroporation Apparatus (Bio-Rad Laboratories, Hercules, CA, USA). The recombinant protein was expressed by adding 1.0 mM IPTG (Promega, Madison, WI, USA) for 3 h at 37 °C, with shaking at 200× *g* per min. Cells were lysed by sonication by applying a continuous pulses of 30 s, with 15 s of interval between them, at 38 MHz, and centrifuged at 13,000*×* g for 20 min at 4 °C. The purified protein was passed on a 5 mL HIS-Trap column (GE Healthcare Life Science) attached to an FPLC (GE Healthcare Life Science) system, and thus through a polymyxin-agarose column (Sigma), in order to remove residual endotoxin content. Aiming to evaluate the purity of the recombinant protein, bacterial total extracts before and after IPTG induction, as well as the purified recombinant protein (10 µg each) were submitted to a 10% SDS-PAGE.

### 4.9. Immunoblotting Assays

To verify the reactivity of the recombinant protein with the specific monoclonal antibodies, β-tubulin (10 µg) was submitted to a 10% SDS-PAGE and blotted onto a nitrocellulose membrane (0.2 μm pore size, Sigma, USA). Then, membranes were blocked with a solution composed by PBS 1× plus Tween 20 0.05% (PBS-T) added with 5% albumin solution and incubated for 1 h at 37 °C. After, they were washed in PBS-T and incubated with the anti-scFv monoclonal antibodies (1:100 diluted in PBS-T) for 1 h at 37 °C. Membranes were washed with PBS-T and the anti-HA peroxidase conjugated antibody (1:5000 diluted in PBS-T) was added, at which time a new incubation was developed for 1 h at 37 °C. As control, an anti-Histidine monoclonal antibody (1:5000 diluted; catalog SAB4301134, Sigma-Aldrich, USA) and the anti-IgG rabbit peroxidase conjugated antibody (1:10,000 diluted in PBS-T; catalog A0545; Sigma-Aldrich, USA) were used. Reactions were developed by adding 12.5 mg chloronaphtol, 25.0 mg diaminobenzidine, and 20 µL H_2_O_2_ 30 vol., and stopped by adding 10 mL distilled water.

### 4.10. Sera and ELISA Assays

Sera samples from VL patients (*n* = 40; including 24 males and 16 females, with ages ranging from 23 to 59 years), of non-infected individuals living in an endemic area of disease (*n* = 40; including 28 males and 12 females, with ages ranging from 25 to 54 years), sera from patients with TL (*n* = 40; including 25 males and 15 females, with ages ranging from 24 to 62 years), Chagas disease (*n* = 30; including 18 males and 12 females, with ages ranging from 31 to 63 years) and leprosy (*n* = 10; including 6 males and 4 females, with ages ranging from 25 to 52 years) were used. The VL diagnosis was confirmed by clinical evaluation and PCR to evaluate the presence of *L. infantum* kDNA. Also, none of the patients had been previously treated with antileishmanial drugs before samples collection. Healthy subjects did not present any clinical signal or suspect of leishmaniasis. Patients with Chagas Disease were confirmed by Chagatest^®^ v.3.0 kit recombinant ELISA kit and Chagatest^®^ hemagglutination inhibition kit (HAI). Leprosy patients were confirmed by clinical evaluation, immunological assays, and DNA detection through RT-PCR. For the ELISA experiments, 96-well plates (Nunc, Nunclon^®^, St. Louis, MO, USA) were sensitized with the recombinant β-tubulin protein or *L. infantum* SLA (0.12 and 1.0 μg/well, respectively), which was diluted in carbonate buffer (0.16% Na_2_CO_3_ and 0.24% NaHCO_3_, pH 9.6), for 16 h at 4 °C. After sensitization, plates were blocked with PBS 1× and BSA 5% (Invitrogen, Sao Paulo, Brazil) for 1 h at 37 °C. After, sera samples (1:200 diluted in PBS 1× and BSA 0.5%) were added to the wells, and the incubation occurred for 1 h at 37 °C. Then, plates were washed 5 times with washing solution (PBS 1× and Tween 20 0.05%) and the anti-human IgG peroxidase antibody (Sigma-Aldrich, Saint Louis, USA), 1:5000 diluted in 1× PBS/0.5% BSA, was added. The incubation was processed for 1 h at 37 °C. After, plates were washed 5 times with the washing solution, and reactions were developed by using 0.1 M citric acid, 0.2 M Na_2_PO_4_, 0.05% OPD and 0.1% H_2_O_2_; and stopped by addition of H_2_SO_4_ 2 M. The optical densities were determined in an ELISA reader (Molecular Devices, Sunnyvale, San Jose, CA, USA) at 492 nm.

### 4.11. In Vitro PBMCs Culture and Cytokine Production

To evaluate the specific cytokine production, blood samples were collected from treated untreated or VL patients (*n* = 10) and healthy subjects (*n* = 10). Patients (*n* = 10, including 5 males and 5 females, with ages ranging from 27 to 56 years) were submitted to the same therapeutic regimen by using a dose of 20 mg Sb^+5^ per kg during 30 days. None of them suffered from any other infections or had any pre-existing disease. PBMCs were purified by density centrifugation through Ficoll-Hypaque (GE Healthcare Bio-Sciences AB, Uppsala, Sweden), according described [47]. Cells (10^7^) were cultured in complete RPMI 1640 medium, which was composed by the medium plus 20% inactivated fetal bovine serum (FBS, Sigma-Aldrich, USA), 2 mM l-glutamine, 200 U/mL penicillin, 100 µg/mL streptomycin, 50 μM 2-mercaptoethanol, 1 mM sodium pyruvate, and 1× non-essential amino acid, and they were incubated in medium (background control) or stimulated with B10, C01 or irrelevant (NRP) phage clones (10^10^ phages, each), with the B10 or C01 peptides (10 µg/mL each), or with β-tubulin and *L. infantum* SLA (10 and 25 µg/mL, respectively), for 5 days at 37°C in 5% CO_2_. The supernatants were collected, and IFN-γ and IL-10 levels were evaluated by capture ELISA by using commercial kits (Human IFN-γ and IL-10 ELISA Sets, BD Biosciences, San Jose, CA, USA), according to manufacturer’s instructions. Results were interpolated from a standard curve using recombinant cytokines (in pg/mL).

### 4.12. Statistical Analysis

The results were analyzed by GraphPad Prism^TM^ (version 5.0 for Windows). Statistical analyses were performed by two-way analysis of variance (ANOVA), followed by Bonferroni’s post-test for comparisons among the groups. Differences were considered significant when *p* < 0.05.

## Supplementary Materials

Supplementary materials can be found at https://www.mdpi.com/1422-0067/20/8/1812/s1.
