# Supplementary material for: Leishmania infantum β-Tubulin Identified by Reverse Engineering Technology through Phage Display Applied as Theranostic Marker for Human Visceral Leishmaniasis
Source: Int J Mol Sci. 2019 Apr 12;20(8):1812. doi: 10.3390/ijms20081812 (PMC6514782; doi:10.3390/ijms20081812)
Supplement: Supplementary file 1 [file ijms-20-01812-s001.pdf]

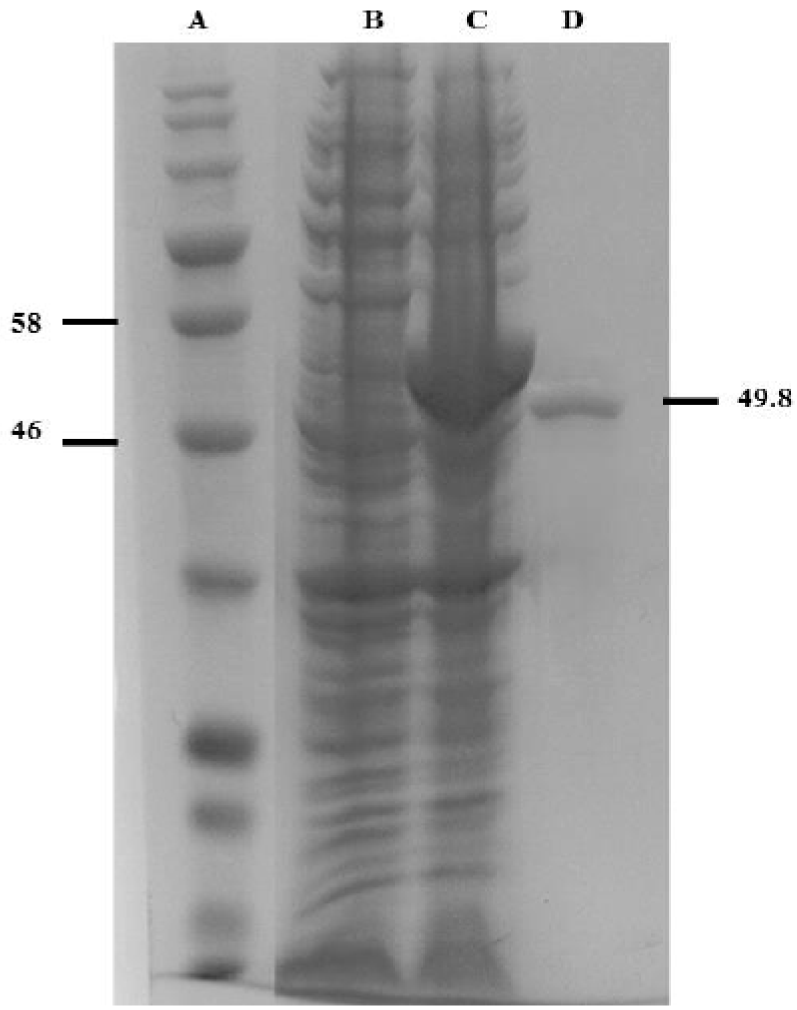

Figure S1. Purification of the recombinant β-tubulin protein. The evaluation of the purification process was obtained after performing a 10% SDS-PAGE gel. In (A), a molecular weight marker with some bands is shown. In (B) and (C), 10 μg of total bacterial cultures non-expressing and expressing the recombinant protein (presenting about 49.8 kDa), respectively, and in (D), the recombinant protein (10 μg) purified by affinity chromatography are also shown.
